# Nirmatrelvir/ritonavir treatment in SARS-CoV-2 positive kidney transplant recipients – a case series with four patients

**DOI:** 10.1186/s12882-023-03154-w

**Published:** 2023-04-15

**Authors:** Johanna Schneider, Rika Wobser, Wolfgang Kühn, Dirk Wagner, Yakup Tanriver, Gerd Walz

**Affiliations:** 1 Department of Medicine IV, Faculty of Medicine, University Freiburg Medical Centre, University of Freiburg, Hugstetter Street 55, Freiburg, 79106 Germany; 2 Department of Medicine II, Division of Infectious Diseases, Faculty of Medicine, University Freiburg Medical Centre, University of Freiburg, Freiburg, Germany

**Keywords:** Nirmatrelvir/ritonavir, Kidney transplantation, COVID-19, CYP3A4 interaction, Case series

## Abstract

**Background:**

Despite vaccination coronavirus disease 2019 (COVID-19)-associated mortality caused by severe acute respiratory syndrome coronavirus 2 (SARS-CoV-2) remains high in kidney transplant recipients. Nirmatrelvir is a protease inhibitor with activity against SARS-CoV-2. Nirmatrelvir reduces the risk for mortality and hospitalization, which is approved for treating adults at risk for severe disease. Nirmatrelvir is metabolized by the cytochrome P-450 (CYP) 3A4 isozyme CYP3A4 and is therefore co-administered with the irreversible CYP3A4 inhibitor ritonavir, which results in a drug interaction with tacrolimus. A limited number of patients with nirmatrelvir/ritonavir and tacrolimus therapy after kidney transplantation have been reported to date. It has been reported that tacrolimus was paused during the five-day nirmatrelvir/ritonavir therapy and subtherapeutic tacrolimus levels were observed after finishing nirmatrelvir/ritonavir in two patients. Therefore, optimization of tacrolimus dosing is urgently needed in transplant recipients receiving nirmatrelvir/ritonavir treatment.

**Case presentation:**

Here, we present our first-hand experience with four patients receiving tacrolimus therapy following kidney transplantation and nirmatrelvir/ritonavir therapy due to COVID-19. Tacrolimus was paused during nirmatrelvir/ritonavir therapy in all patients, which resulted in stable therapeutic tacrolimus levels. Tacrolimus was continued directly after finishing nirmatrelvir/ritonavir to avoid subtherapeutic levels in the first patient treated. This patient received his usual tacrolimus maintenance dose, which resulted in toxic levels. Based on this observation, tacrolimus therapy was continued 24 h after finishing nirmatrelvir/ritonavir treatment at a reduced dose in the subsequent patients. In these patients, therapeutic to supratherapeutic tacrolimus levels were observed despite the therapeutic break and dose reduction.

**Discussion and conclusions:**

Based on altered CYP3A4 metabolism, tacrolimus levels have to be closely monitored after treatment with nirmatrelvir/ritonavir. Our study suggests that tacrolimus treatment should be paused during nirmatrelvir/ritonavir medication and be continued 24 h after completing nirmatrelvir/ritonavir therapy at a reduced dose and under close monitoring. Based on the limited number of patients in this study, results must be interpreted with caution.

## Background

The risk for Coronavirus disease 2019 (COVID-19)-associated mortality and hospital admission is eightfold increased in kidney transplant recipients compared to the general population [1]. Despite vaccination, COVID-19 mortality rates in solid organ transplant recipients remain high with 8% compared to 10% in unvaccinated patients [2]. Risk factors for mortality in these patients comprise age, obesity, transplantation with a deceased donor, diabetes, cardiovascular and respiratory diseases [3, 4]. The increased mortality is at least in part due to the impaired immunological response with adequate antibody titres in only about 34–54% after the second severe acute respiratory syndrome coronavirus 2 (SARS-CoV-2) vaccination [5, 6]. Consequently, breakthrough infections after two doses of vaccination are more prevalent, affecting 8% of kidney transplant recipients [7]. Thus, there is an urgent need to optimize COVID-19 treatment of this high-risk patient population. Since mycophenolate treatment increases mortality in kidney transplant recipients with COVID-19 [8], mycophenolate is typically discontinued during SARS-CoV2 infection in these patients [9]. However, mycophenolate dose reduction within the first year following kidney transplantation increases the incidence of acute rejection and impairs graft survival [10]. Of note, interrupting mycophenolate therapy for only 6 days increases rejection rates following kidney transplantation [11].

In January 2022, oral therapy with nirmatrelvir/ritonavir against SARS-CoV-2 was approved by the European Medicines Agency for treating COVID-19 in adults at increased risk for developing severe disease. Nirmatrelvir inhibits the SARS-CoV-2 3-chymotrypsin-like cysteine protease [12], which reduced the relative risk of mortality and hospitalization by 89% in a phase 2–3 trial [13]. Nirmatrelvir is metabolized by the cytochrome P-450 (CYP) 3A4 isozyme (CYP3A4), and is therefore co-administered with the irreversible CYP3A4 inhibitor ritonavir. Tacrolimus is a standard immunosuppressive agent used in organ transplant recipients that is also metabolized by CYP3A4. Therefore, a pronounced drug interaction between tacrolimus and nirmatrelvir/ritonavir is to be expected, warranting close monitoring and further investigation. Lange et al. suggest to pause tacrolimus, to check tacrolimus levels 3 days later, and to restart tacrolimus after completion of the nirmatrelvir/ritonavir course at a reduced dose of 25–50% compared to baseline after tacrolimus levels reach therapeutic levels [14]. These recommendations emanate from the experience with combined treatment of ritonavir with anti-hepatitis C virus (HCV) drugs. Wang et al. tested this protocol in four kidney transplant patients. The authors observed stable tacrolimus levels during treatment with nirmatrelvir/ritonavir; however, subtherapeutic levels were observed in two patients 2–3 days after finishing nirmatrelvir/ritonavir treatment [15]. Therefore, optimization of tacrolimus dosing is urgently needed in transplant recipients receiving nirmatrelvir/ritonavir for treatment of COVID-19. Here, we report the tacrolimus levels of four kidney transplant recipients that received nirmatrelvir/ritonavir to prevent severe COVID-19 disease.

## Case presentation

We retrospectively analysed the first four kidney transplant patients with COVID-19, who received treatment with nirmatrelvir/ritonavir at the Medical Centre of the University of Freiburg, Germany. Written informed consent was obtained from all patients presented. The need for an ethics approval was waived by the ethics committee of the Freiburg Medical Centre. Clinical data were collected from patient records. All patients were in inpatient care. GraphPad™ Prism Version 8 software (GraphPad Software, San Diego, CA) was used for diagram preparation.

Patients #1, #2 and #4 were > 1-year post-transplantation (Table 1). Patient #3 received an ABO-incompatible kidney living donation four months before COVID-19 infection. Patient #1 received a kidney transplantation from a deceased donor, patient #2 received an ABO-compatible living kidney donation, and patient #4 an ABO-incompatible living kidney donation (Table 1). All patients received tacrolimus immediate release, and displayed stable tacrolimus levels and eGFR ≥ 30 mL/min/1.73m^2^ prior to SARS-CoV-2 infection.

**Table 1:** Baseline characteristics and clinical course of patients

**Patient**	**Age** (years)	**Kidney transplant** (years prior to COVID-19)	**Concomitant diseases**	**Induction therapy**	**Immunosuppressive medication**	**Tacrolimus level prior to COVID-19** (ng/mL)	**eGFR prior to COVID-19** (mL/min/1.73 m^2^)	**Vaccination status** (number of vaccines received)	**SARS-CoV2 S1-IgG** (BAU/mL)	**Symptoms**	**NEWS 2**	**Oxygen dependency**	**COVID-19 treatment other than nirmatrelvir / ritonavir**	**Clinical course and complications**
1	74	Deceased donor (3)	ADPKD, hypertensive heart disease, aortic regurgitation, cirrhosis CHILD–Pugh class A, PTDM	basiliximab	tacrolimus, azathioprine, corticosteroid	4.9	55	3	171	fatigue	4	no	sotrovimab	cholangiosepsis, death
2	62	ABOc living donation (5)	IgA nephropathy, PTDM, fatty liver, transplant renal artery stenosis	basiliximab	tacrolimus, mycophenolate, corticosteroid	5.5	40	4	negative	fatigue, fever, coughing	4	no	sotrovimab	improvement, no complications
3	25	ABOi living donation (0)	posterior urethral valves, hypertension	rituximab, thymoglobulin	tacrolimus, mycophenolate, corticosteroid	6.5	55	3	> 2180	fatigue, fever, coughing, sore throat	3	no	no	improvement, no complications
4	60	ABOi living donation (4)	hypertension, hypertensive heart disease, PTDM	rituximab, basiliximab	tacrolimus, mycophenolate, corticosteroid	6.4	45	3	negative	fatigue, diarrhoea, coughing, vomiting, weight loss, hyponatremia, dehydration	12	max.8 L/min	sotrovimab, tocilizumab	improvement, no complications

*ABOc* Blood group compatible, *ABOi* Blood group incompatible, *ADPKD* autosomal dominant polycystic kidney disease, *BAU* Binding antibody units, *COVID-19* Coronavirus disease 2019, *eGFR* Estimated glomerular filtration rate, *IgG* Immunoglobulin G, *NEWS 2* National Early Warning Score 2*, PTDM* Post-transplantation diabetes mellitus, *SARS-CoV2* Severe acute respiratory syndrome coronavirus 2; S1, subunit of spike protein of SARS-CoV

**Fig. 1:**
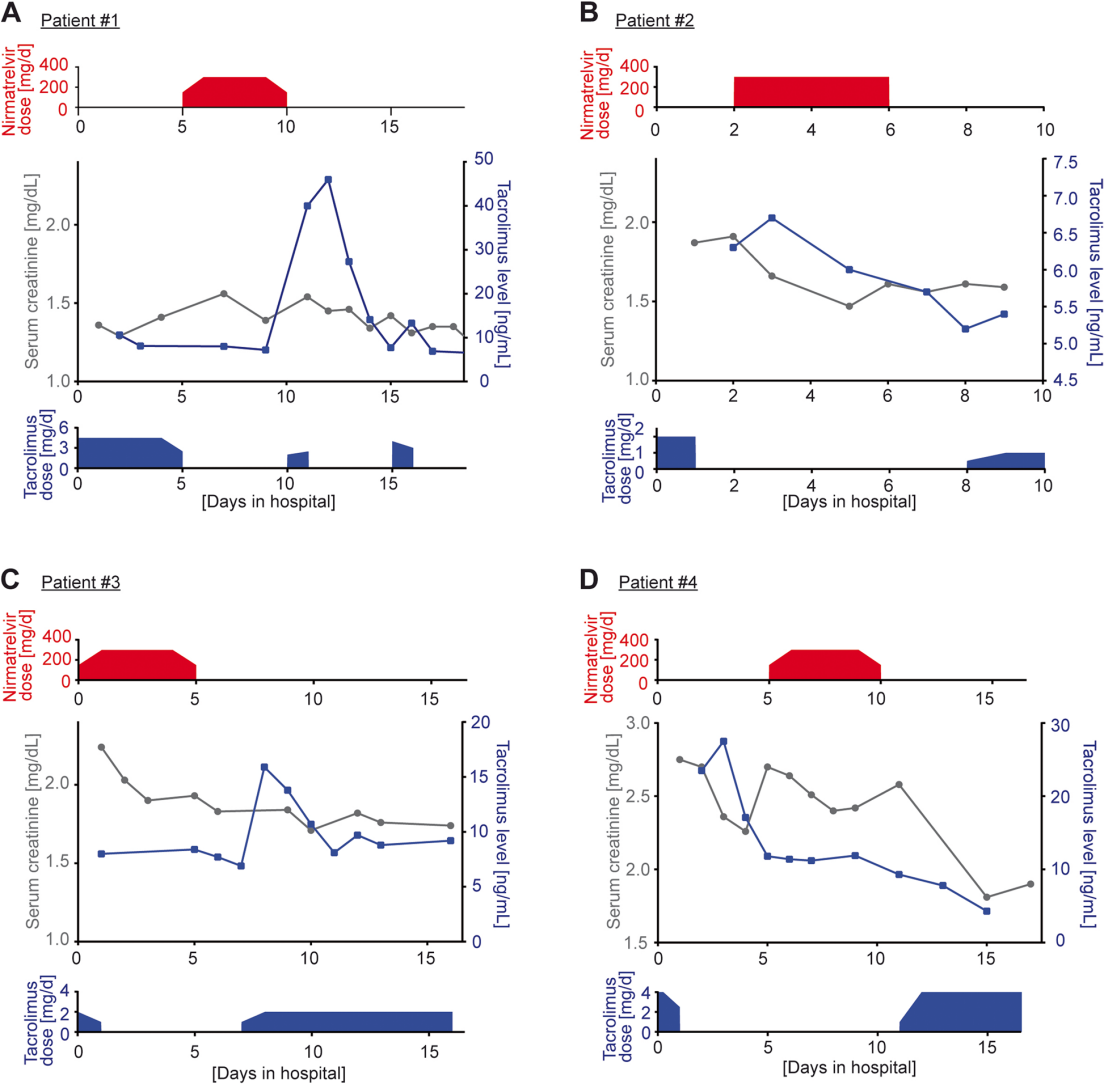
Time course of nirmatrelvir dose, serum creatinine levels, tacrolimus levels, and tacrolimus dose

In patient #1, maintenance immunosuppressive therapy comprised tacrolimus, azathioprine, and corticosteroids; patient #2–4 received tacrolimus, mycophenolate, and corticosteroids (Table 1). In all patients, mycophenolate and azathioprine were withdrawn during nirmatrelvir/ritonavir treatment. Patient #2 had received corticosteroid pulse therapy due to rejection five years prior to COVID-19. Since all patients exhibited an eGFR < 60 mL/min/1.73 m^2^ (Table 1), patients received a reduced nirmatrelvir dose of 150 mg twice a day (Fig. 1). Nirmatrelvir/ritonavir treatment was started between day 1–5 after hospital admission. In patient #4, treatment was started on day five due to initial supratherapeutic tacrolimus levels, which were likely caused by COVID-19 associated diarrhoea. National Early Warning Score (NEWS) 2 is used to assess severity of disease of COVID-19 patients [16]. Patient #4 additionally received the anti-interleukin-6 receptor antibody tocilizumab due to a severe course of disease with a NEWS 2 of 12 and a maximum oxygen requirement of 8 L/min (Table 1). Patients #1, #2 and #3 had a NEWS 2 of 3–4 (Table 1), which indicates a low clinical risk. These patients did not require oxygen supply. All patients had received at least three vaccinations against COVID-19 prior to infection (Table 1). Patient #2 and #4 did not respond to vaccination as indicated by negative SARS-CoV2 S1-IgG titres; patient #1 exhibited low SARS-CoV2 S1-IgG titres. Patient #1, #2 and #4 also received the SARS-CoV-2 neutralizing antibody sotrovimab due to an insufficient response to previous SARS-CoV-2 vaccinations (Table 1). In all patients, tacrolimus treatment was stopped at the day of nirmatrelvir/ritonavir initiation. Tacrolimus level was 4.9–6.5 ng/mL prior to nirmatrelvir/ritonavir treatment (Table 1) and was stable between 6–11.9 ng/mL during nirmatrelvir/ritonavir treatment (Fig. 1). To avoid drug interactions in addition to nirmatrelvir/ritonavir and tacrolimus, no drug listed in the manufacturers’ product information was administered simultaneously.

In patient #1, tacrolimus was started with the initial dose after finishing the five-day course of nirmatrelvir/ritonavir. The patient received two doses of tacrolimus, which resulted in a toxic peak level of 46 ng/mL. Tacrolimus was discontinued for three days until levels declined to 7.7 ng/mL. A reduced tacrolimus dose for the duration of one day increased levels to 13.3 ng/mL the following day. Due to cholangiosepsis, immunosuppressive therapy including tacrolimus was then switched to hydrocortisone as continuous infusion, as previously described [17]. The patient died 46 days after the diagnosis of COVID-19. In patient #2, tacrolimus was restarted one day after finishing nirmatrelvir/ritonavir treatment at a reduced dose of 50%, which resulted in stable tacrolimus levels the following three days. In patient #3, tacrolimus therapy was initiated the day after completing nirmatrelvir/ritonavir treatment. Despite a 50% dose reduction, tacrolimus levels increased from 6.9 ng/mL to a maximum of 15.9 ng/mL. The patient received his baseline dose five days after finishing nirmatrelvir/ritonavir that resulted in a peak level of 9.7 ng/mL. Patient #4 received a 11% reduced dose of tacrolimus 24 h after finishing nirmatrelvir/ritonavir with stable tacrolimus levels between 4.3–7.8 ng/mL during the following five days. Patient #2–4 recovered from COVID-19 with no residual complications.

## Discussion and conclusions

Here, we present the results from four patients with kidney transplantation and nirmatrelvir/ritonavir treatment. Since the randomized Evaluation of Protease Inhibition for COVID-19 in High-Risk Patients (EPIC-HR) trial showed a reduced risk for hospitalization and death in adults with at least one risk factor for a severe course of the disease [18], nirmatrelvir/ritonavir received a conditional license in Germany in January 2022 and treatment was used in the patients reported. Remdesivir treatment reduced the risk of hospitalization, but not death, in a randomized controlled trial, which comprised patients with a high risk for progression to severe disease [19]; only recent data from the Lean European Open Survey showed a reduced mortality for patients starting remdesivir administration in the complicated phase of disease, e.g. mainly needing oxygen supplementation [20]. Nirmatrelvir/ritonavir was therefore preferred over remdesivir for the patients discussed in the present report. Sotrovimab treatment is safe for patients with solid organ transplantation [21, 22] and attenuated the course of severe disease relative to controls. Omicron BA.2 was the main SARS-CoV-2 variant in Germany at the time of treatment of the patients reported [23]. Even though sotrovimab has an reduced neutralizing activity against Omicron BA.2 [24], these data were not available at the time of treatment. We thus administered sotrovimab to our patients with no or limited response to vaccination, i.e., patient #1, #2 and #4. Studies investigating the therapeutic effect of molnupiravir do not consistently demonstrate efficacy against hospitalization or death. Therefore, the substance was recommended as second choice compared to nirmatrelvir/ritonavir [25, 26] and was not used in our patients. In fact, the European Medicines Agency (EMA) just recently has refused to recommend the approval of molnupiravir since clinical benefit in adults with COVID-19 who are not on supplemental oxygen and at increased risk of developing severe COVID-19 could not be demonstrated.

All patients in the present study and the report by Wang et al. [15] showed therapeutic or supratherapeutic tacrolimus levels during nirmatrelvir/ritonavir treatment despite discontinuation of tacrolimus treatment. The present report supports the recommendation by Wang et al. to withhold tacrolimus during nirmatrelvir/ritonavir treatment. Experiences with ritonavir-boosted antiviral therapy over longer time periods suggest that a substantial reduction in the tacrolimus dose is required to reach acceptable tacrolimus levels. In a study investigating the combined therapy of ritonavir and antiviral HCV therapy, tacrolimus was administered at a dose of 0.2–0.5 mg every 5–10 days [27]. Similarly, therapeutic tacrolimus levels were reported for HIV-positive patients that were treated with ritonavir-boosted antiviral therapy at a dose of 0.03–0.08 mg/d [28].

Ritonavir significantly prolongs the half-life of tacrolimus as indicated by an increase from 32 to 232 h in healthy volunteers [29] and thus, it provides a challenge to determine when to restart tacrolimus therapy. Wang et al. observed subtherapeutic tacrolimus levels at day 8 and 9, which resulted in the suggestion to reintroduce the full or reduced dose between day 8 and 10 [15]. This contrasts to our patients, in which tacrolimus was re-introduced earlier to avoid subtherapeutic levels. Patient #1 was the first kidney transplant recipient treated with nirmatrelvir/ritonavir at our centre. In this patient, tacrolimus was started immediately after completing the five-day nirmatrelvir/ritonavir course at the normal dose. Tacrolimus levels reached 40 ng/mL after a single dose of 2 mg and further increased to 46 ng/mL after another dose of 2.5 mg, which corresponds to his baseline doses. Although tacrolimus treatment was stopped immediately, levels remained elevated, declining to 7.7 ng/mL 3.5 days after the second dose. This underlines the need for a dose reduction when re-introducing tacrolimus. Based on the experience with patient #1, tacrolimus was restarted in patients #2, #3 and #4 24 h after the five-day nirmatrelvir/ritonavir course, and the dose was reduced compared to the baseline dose by 50% in patient #2 and #3 and by 11% in patient #4. This resulted in stable tacrolimus levels in patient #2 and #4. Patient #3 had a supratherapeutic level with 15.9 mg/mL; subtherapeutic levels were not observed.

Based on altered CYP3A4 metabolism and individual differences in tacrolimus metabolism, serum levels have to be closely monitored in kidney transplant recipients during and especially after treatment with nirmatrelvir/ritonavir. The present study suggests that tacrolimus treatment should be temporarily paused during nirmatrelvir/ritonavir treatment to avoid supratherapeutic levels. However, it should be noted that only a small number of patients was examined. Re-introducing tacrolimus therapy after nirmatrelvir/ritonavir can be challenging. The present study suggests that it might be an option to start tacrolimus 24 h after completing nirmatrelvir/ritonavir therapy at a reduced dose and under close monitoring. While COVID-19 positive kidney transplant recipients might benefit from nirmatrelvir/ritonavir therapy, the present study indicates that additional studies with greater patient numbers are required to optimize the therapeutic immunosuppressive management.

## Data Availability

All data generated or analyzed during this study are included in this published article.

## Abbreviations

ABOi: Blood group incompatible
COVID-19: Coronavirus disease 2019 caused by severe acute respiratory syndrome coronavirus
CYP3A4: Cytochrome P-450 (CYP) 3A4 isozyme
EMA: European Medicines Agency
EPIC-HR: Evaluation of Protease Inhibition for COVID-19 in High-Risk Patients
HCV: Hepatitis C virus
NEWS 2: National Early Warning Score 2
SARS-CoV-2: Severe acute respiratory syndrome coronavirus 2

## References

[1] Hippisley-Cox J, Coupland CA, Mehta N, Keogh RH, Diaz-Ordaz K, Khunti K (2021). Risk prediction of covid-19 related death and hospital admission in adults after covid-19 vaccination: national prospective cohort study. BMJ.

[2] Callaghan CJ, Mumford L, Curtis RMK, Williams SV, Whitaker H, Andrews N (2022). Real-world Effectiveness of the Pfizer-BioNTech BNT162b2 and Oxford-AstraZeneca ChAdOx1-S Vaccines Against SARS-CoV-2 in Solid Organ and Islet Transplant Recipients. Transplantation.

[3] Kates OS, Haydel BM, Florman SS, Rana MM, Chaudhry ZS, Ramesh MS, et al. Coronavirus Disease 2019 in Solid Organ Transplant: A Multicenter Cohort Study. Clin Infect Dis. 2021;73(11):e4090–9. Pubmed Central PMCID: 7454362. 10.1093/cid/ciaa1097 PMC7454362 32766815

[4] Udomkarnjananun S, Kerr SJ, Townamchai N, Susantitaphong P, Tulvatana W, Praditpornsilpa K (2021). Mortality risk factors of COVID-19 infection in kidney transplantation recipients: a systematic review and meta-analysis of cohorts and clinical registries. Sci Rep.

[5] Boyarsky BJ, Werbel WA, Avery RK, Tobian AAR, Massie AB, Segev DL (2021). Antibody Response to 2-Dose SARS-CoV-2 mRNA Vaccine Series in Solid Organ Transplant Recipients. JAMA.

[6] Marion O, Del Bello A, Abravanel F, Couat C, Faguer S, Esposito L (2021). Safety and Immunogenicity of Anti-SARS-CoV-2 Messenger RNA Vaccines in Recipients of Solid Organ Transplants. Ann Intern Med.

[7] Bell S, Campbell J, Lambourg E, Watters C, O’Neil M, Almond A, et al. The Impact of Vaccination on Incidence and Outcomes of SARS-CoV-2 Infection in Patients with Kidney Failure in Scotland. J Am Soc Nephrol. 2022;33(4):677–86. (PubMed PMID: 35110363). 10.1681/ASN.2022010046 PMC8970454 35110363

[8] Requiao-Moura LR, Modelli de Andrade LG, de Sandes-Freitas TV, Cristelli MP, Viana LA, Nakamura MR, et al. The Mycophenolate-based Immunosuppressive Regimen Is Associated With Increased Mortality in Kidney Transplant Patients With COVID-19. Transplantation. 2022;106(10):e441–51. PubMed PMID: 35765133. Pubmed Central PMCID: 9521389. 10.1097/TP.0000000000004251 PMC9521389 35765133

[9] Caillard S, Anglicheau D, Matignon M, Durrbach A, Greze C, Frimat L (2020). An initial report from the French SOT COVID Registry suggests high mortality due to COVID-19 in recipients of kidney transplants.. Kidney Int.

[10] Pelletier RP, Akin B, Henry ML, Bumgardner GL, Elkhammas EA, Rajab A (2003). The impact of mycophenolate mofetil dosing patterns on clinical outcome after renal transplantation. Clin Transplant.

[11] Zafrani L, Truffaut L, Kreis H, Etienne D, Rafat C, Lechaton S (2009). Incidence, risk factors and clinical consequences of neutropenia following kidney transplantation: a retrospective study. Am J Transplant Off J Am Soc Transplant Am Soc Transplant Surg.

[12] Owen DR, Allerton CMN, Anderson AS, Aschenbrenner L, Avery M, Berritt S (2021). An oral SARS-CoV-2 M(pro) inhibitor clinical candidate for the treatment of COVID-19. Science.

[13] Hammond J, Leister-Tebbe H, Gardner A, Abreu P, Bao W, Wisemandle W (2022). Oral Nirmatrelvir for High-Risk, Nonhospitalized Adults with Covid-19. N Engl J Med.

[14] Lange NW, Salerno DM, Jennings DL, Choe J, Hedvat J, Kovac DB, et al. Nirmatrelvir/ritonavir use: Managing clinically significant drug-drug interactions with transplant immunosuppressants. American journal of transplantation : official journal of the American Society of Transplantation and the American Society of Transplant Surgeons. 2022 Jan 11. PubMed PMID: 35015924. 10.1111/ajt.16955 35015924

[15] Wang AX, Koff A, Hao D, Tuznik NM, Huang Y. Effect of nirmatrelvir/ritonavir on calcineurin inhibitor levels: Early experience in four SARS-CoV-2 infected kidney transplant recipients. American journal of transplantation : official journal of the American Society of Transplantation and the American Society of Transplant Surgeons. 2022 Feb 14. PubMed PMID: 35158412. 10.1111/ajt.16997 PMC9111225 35158412

[16] Kostakis I, Smith GB, Prytherch D, Meredith P, Price C, Chauhan A (2021). The performance of the National Early Warning Score and National Early Warning Score 2 in hospitalised patients infected by the severe acute respiratory syndrome coronavirus 2 (SARS-CoV-2). Resuscitation.

[17] Henningsen M, Jaenigen B, Zschiedrich S, Pisarski P, Walz G, Schneider J (2021). Risk Factors and Management of Leukopenia After Kidney Transplantation: A Single-Center Experience. Transpl Proc.

[18] Hammond J, Leister-Tebbe H, Gardner A, Abreu P, Bao W, Wisemandle W, et al. Oral Nirmatrelvir for High-Risk, Nonhospitalized Adults with Covid-19. The New England journal of medicine. 2022 Feb 16. PubMed PMID: 35172054. Pubmed Central PMCID: 8908851. 10.1056/NEJMoa2118542 PMC8908851 35172054

[19] Gottlieb RL, Vaca CE, Paredes R, Mera J, Webb BJ, Perez G (2022). Early Remdesivir to Prevent Progression to Severe Covid-19 in Outpatients. N Engl J Med.

[20] Pilgram L, Appel KS, Ruethrich MM, Koll CEM, Vehreschild M, de Miranda SMN, et al. Use and effectiveness of remdesivir for the treatment of patients with covid-19 using data from the Lean European Open Survey on SARS-CoV-2 infected patients (LEOSS): a multicentre cohort study. Infection. 2023:1–17. PubMed PMID: 36763285. Pubmed Central PMCID: 9913009. 10.1007/s15010-023-01994-0 PMC9913009 36763285

[21] Pinchera B, Buonomo AR, Scotto R, Carrano R, Salemi F, Galluccio F, et al. Sotrovimab in Solid Organ Transplant Patients With Early, Mild/Moderate SARS-CoV-2 Infection: A Single-center Experience. Transplantation. 2022;106(7):e343–5. PubMed PMID: 35349534. Pubmed Central PMCID: 9213056. 10.1097/TP.0000000000004150 PMC9213056 35349534

[22] Chavarot N, Melenotte C, Amrouche L, Rouzaud C, Sberro-Soussan R (2022). Early treatment with sotrovimab monoclonal antibody in kidney transplant recipients with Omicron infection. Kidney Int.

[23] Touret F, Baronti C, Bouzidi HS, de Lamballerie X (2022). In vitro evaluation of therapeutic antibodies against a SARS-CoV-2 Omicron B.1.1.529 isolate. Sci Rep.

[24] Takashita E, Yamayoshi S, Simon V, van Bakel H, Sordillo EM, Pekosz A, et al. Efficacy of Antibodies and Antiviral Drugs against Omicron BA.2.12.1, BA.4, and BA.5 Subvariants. The New England journal of medicine. 2022;387(5):468–70. PubMed PMID: 35857646. Pubmed Central PMCID: 9342381. 10.1056/NEJMc2207519 PMC9342381 35857646

[25] Jayk Bernal A, Gomes da Silva MM, Musungaie DB, Kovalchuk E, Gonzalez A, Delos Reyes V (2022). Molnupiravir for Oral Treatment of Covid-19 in Nonhospitalized Patients. N Engl J Med.

[26] Butler CC, Hobbs FDR, Gbinigie OA, Rahman NM, Hayward G, Richards DB (2023). Molnupiravir plus usual care versus usual care alone as early treatment for adults with COVID-19 at increased risk of adverse outcomes (PANORAMIC): an open-label, platform-adaptive randomised controlled trial. Lancet.

[27] Kwo PY, Mantry PS, Coakley E, Te HS, Vargas HE, Brown R (2014). An interferon-free antiviral regimen for HCV after liver transplantation. N Engl J Med.

[28] Bickel M, Anadol E, Vogel M, Hofmann WP, von Hentig N, Kuetscher J, et al. Daily dosing of tacrolimus in patients treated with HIV-1 therapy containing a ritonavir-boosted protease inhibitor or raltegravir. The Journal of antimicrobial chemotherapy. 2010;65(5):999–1004. PubMed PMID: 20202988. Pubmed Central PMCID: 2902821. 10.1093/jac/dkq054 PMC2902821 20202988

[29] Badri P, Dutta S, Coakley E, Cohen D, Ding B, Podsadecki T, et al. Pharmacokinetics and dose recommendations for cyclosporine and tacrolimus when coadministered with ABT-450, ombitasvir, and dasabuvir. American journal of transplantation : official journal of the American Society of Transplantation and the American Society of Transplant Surgeons. 2015;15(5):1313–22. PubMed PMID: 25708713. Pubmed Central PMCID: 5024008. 10.1111/ajt.13111 PMC5024008 25708713

